# Access to after-hours primary care: a key determinant of children’s medical home status

**DOI:** 10.1186/s12913-021-06192-y

**Published:** 2021-02-27

**Authors:** Bing Han, Peggy Guey-Chi Chen, Hao Yu

**Affiliations:** 1 Health Unit, RAND Corporation, 1776 Main Street, Santa Monica, CA 90401 USA; 2 Department of Population Medicine, Harvard Medical School and Harvard Pilgrim Health Care Institute, Landmark Center, 401 Park Drive, Suite 401 East, Boston, MA 02215 USA

**Keywords:** Medical home, Accessibility, Marker, Child, After-hours access

## Abstract

**Background:**

The medical home (MH) model has been promoted by both the federal and state governments in the United States in recent years. To ascertain American children’s MH status, many studies have relied on a large set of survey items, posing a considerable burden on their parents. We aimed to identify individual survey items or domains that best predict MH status for children and use them to develop brief markers of MH status. We also examined whether the identified items differed by status of special health care needs and by racial/ethnic group.

**Method:**

Using the 9-year data from Medical Expenditure Panel Survey, we examined associations between children’s MH status and individual survey items or domains. We randomly split the data into two halves with the first half (*training sample*, *n* = 8611) used to identify promising items, and the second half (*validation sample*, *n* = 8779) used to calculate all statistical measures. After discovering significant predictors of children’s MH status, we incorporated them into several brief markers of MH status. We also conducted stratified analyses by status of special health care needs and by racial/ethnic group.

**Results:**

Less than half (48.7%) of the 8779 study children had a MH. The accessibility domain has stronger association with children’s MH status (specificity = 0.84, sensitivity = 1, Kappa = 0.83) than other domains. The top two items with the strongest association with MH status asked about after-hours primary care access, including doctors’ office hours at night or on the weekend and children’s difficulty accessing care after hours. Both belong to the accessibility domain and are one of several reliable markers for children’s MH status. While each of the two items did not differ significantly by status of special health care needs, there were considerable disparities across racial/ethnic groups with Latino children lagging behind other children.

**Conclusion:**

Accessibility, especially the ability to access health care after regular office hours, appears to be the major predictor of having a MH among children. The ongoing efforts to promote the MH model need to target improving accessibility of health care after regular hours for children overall and especially for Latino children.

## Background

Recent years have witnessed an increased interest in promoting the pediatric medical home (MH) model, which was defined by the American Academy of Pediatrics (AAP) as “a model of delivering primary care that is *accessible*, *continuous*, *comprehensive*, *family-centered*, *coordinated*, *compassionate*, and *culturally effective* (i.e., the seven defining domains or components of MH) to every child and adolescent [1, 2].” Prior studies found that children with MHs had fewer visits and lower costs to emergency care than children without MHs [3–8]. MH has also been linked to increased preventive visits and dental care visits among children [7], as well as higher ratings of health care quality [6]. One recent systematic review identified generally positive associations between having a MH and various outcomes of child well-being [9].

Despite the growing literature, there is no consensus among researchers about how to empirically define children’s MH status. For example, the National Committee for Quality Assurance has promoted the Patient-Centered MH model by offering different levels of accreditation to physician practices for achieving specific model features [10]. A practice’s accreditation can be used to define MH status for the patients served at the practice [11–13]. Whereas this approach measures MH status from the practice perspective, other studies have defined MH status based on health care experiences [6, 7, 14, 15]. Arguably, patients’ experiences are as important as practice features in measuring the quality of primary care. That is why many studies have used pediatric care experience to define MH status for children. Those studies typically operationalized the AAP definition of MH by using survey data about pediatric care experience [9, 14, 16, 17]. For example, one study examined children’s MH status using the National Survey of Children’s Health [17]. Other studies have used the Medical Expenditures Panel Survey to define children’s MH status [7].

Theoretically, a MH should be defined by all the above seven domains with equal relevance. In empirical studies, however, domains can have very heterogeneous associations with the MH status. For example, one study reported that the family-centered domain had a much stronger association with a child’s MH status than the comprehensiveness domain [17]. While the study represents the first analysis of the heterogeneous associations between individual domains and the overall MH status for a child, further research on this issue is needed for several reasons. First, a better understanding of the specific domains and survey items that are significantly associated with the overall MH status would greatly inform future efforts and resource allocation to study MHs for children. Conversely, domains or survey items without notable associations with the overall MH status may lack variation (i.e. they may be endorsed by most patients), and subsequently will not be useful for future studies of children’s MH statuses. Second, after identifying the survey items with the strongest associations with the overall MH status, they may be used to develop a shorter list of survey items as a brief marker for MH status. A shorter list of survey items would make screening for MH more efficient, compared with the current survey instrument usually consisting of 20 or more survey questions, which can be a significant burden on patients and their families, raising concerns about the scarce resources available for conducting surveys. Finally, beyond a research application, there may also be utility of a marker in a clinical setting by helping identify patients’ needs either at initial patient intake or in an ongoing fashion to ensure that the new needs are addressed.

To examine these questions, we analyzed the association between the overall MH status for a child and individual domains in a nationally representative survey. We identified specific survey items with strong associations with MH status, and used them to explore the development of a short list, or a marker for children’s MH status. We also examined whether the domains or items that were strongly associated with the MH status differed by status of special health care needs and by racial/ethnic group since prior studies indicated that MH status varied by these factors [18–20].

## Methods

### Data

We used the Medical Expenditure Panel Survey (MEPS) from 2004 to 2012. The MEPS is a nationally representative survey conducted by the Agency for Healthcare Research and Quality. Compared to other data sources, such as the National Survey of Children’s Health, MEPS has a much more comprehensive list of survey items on pediatric care experiences. Each MEPS panel consists of a nationally representative sample that is followed-up for 2 years. We used the first year in each panel to avoid the intra-person correlation in all variables.

### MH status

We used the method developed by Romaire and Bell, who used 22 MEPS survey items to define children’s MH status [7]. To have a MH, a child first must have a qualified usual source of care (USC) (See details in Table 1 about the definition of a qualified USC). Then, those children having a qualified USC were scored based on the MEPS survey items (See details in Table 1) in four MH domains (accessible, comprehensive, family-centered, and compassionate)*.* The other three domains of the AAP definition of MH model could not be measured because of inadequate information in the MEPS. Table 1 provides detailed information about the 22 MEPS survey items. The minimum of the four domain scores needed to be no less than the cutoff of 75 points. Missing data in survey items that was not a legitimate skip led to a missing MH status for a child. We identified 17,390 children having sufficient information to apply the MH definition in Table 1.

**Table 1:** MEPS survey items and scoring by MH domain ^a^

Domain and item	Description of MEPS question and scoring
**Usual source of care** ^b^	
USC1	Is there a particular doctor’s office, clinic, health center, or other place that the individual usually goes to if he/she is sick or needs advice about his/her health?
USC2	Is the individual’s provider a person, facility, or person in a facility?
**Accessibility** (average of ACC1 to ACC4)	
ACC1	Does the provider have office hours at night or on the weekend? No (0) Yes (100)
ACC2	Any difficulty accessing care after hours? Very difficult (0) Somewhat difficult (25) Not too difficult (75) Not at all difficult (100)
ACC3	Any difficulty getting to care? Very difficult (0) Somewhat difficult (25) Not too difficult (75) Not at all difficult (100)
ACC4	Any difficulty accessing provider by phone? Very difficult (0) Somewhat difficult (25) Not too difficult (75) Not at all difficult (100)
**Family centered** (average of FAM1 to FAM7)	
FAM1	Provider listened to the parent? Never (0) Sometimes (25) Usually (75) Always (100)
FAM2	Provider spent enough time with the person and parent? Never (0) Sometimes (25) Usually (75) Always (100)
FAM3	Provider seeks the parent’s advice when deciding treatments? No (0) Yes (100)
FAM4	Provider shows respect for treatments other doctors may give? Never (0) Sometimes (25) Usually (75) Always (100)
FAM5	Provider asks the parent to help make decisions? No (0) Yes (100)
FAM6	Provider explains options to the parent? No (0) Yes (100)
FAM7	Provider explains things in a way the parent can understand? Never (0) Sometimes (25) Usually (75) Always (100)
**Comprehensive** (average of CPR2, CPR4, CPR6, and CPR8, excluding legitimate skips defined by CPR1, CPR3, CPR5, and CPR7; domain scored 100 if all items skipped legitimately)	
CPR1	Did the child need to see a specialist?
CPR2	If needed a specialist, any problems accessing specialist? A big problem (0) A small problem (50) Not a problem (100)
CPR3	Did the child have an illness, injury, or condition that needed care right away from a clinic, emergency room, or doctor’s office?
CPR4	If needed care right away, how often was care received as soon as wanted? Never (0) Sometimes (25) Usually (75) Always (100)
CPR5	Were there any appointments made to see a doctor or other health provider for routine health care?
CPR6	If appointments were made for routine care, how often was an appointment secured as soon as wanted? Never (0) Sometimes (25) Usually (75) Always (100)
CPR7	Did the parent or a doctor believe the person needed any care, tests, or treatment?
CPR8	If needed care or treatment, any problems receiving care? A big problem (0) A small problem (50) Not a problem (100)
**Compassionate** (CPA1)	
CPA1	Provider shows respect for what the parent has to say? Never (0) Sometimes (25) Usually (75) Always (100)

^a^ Table reproduced from Romaire and Bell [7]

^b^ USC is the prerequisite of MH status. No USC or no qualified USC results in a MH score of 0 and hence not having a MH

### Item status

Responses to each survey item in Table 1 was assigned a score, ranging from 0 to 100, by using the method in a prior study [7]. Any item on which the individual child scored 75 points or more was considered a positive item status. Otherwise, a negative item status was considered for an item scored less than 75 points. For example, if a child had a response of “sometimes” to question CPA1 “Provider shows respect for what the parent has to say”, then the CPA1 item score was 25, and consequently the CPA1 item status was negative for the child.

### Domain status

The domain score was the average of all the item scores in a domain. Dichotomous domain statuses (positive/negative) was defined using the cutoff of 75 points on domain scores. For example, if a child received item scores of 0, 25, 100, and 100 for the four items under the accessible domain respectively (ACC1-ACC4), then the accessible domain score was 56 and the accessible domain status was negative for the child.

It is worth noting that, for the domain of comprehensive care, it is possible to legitimately skip some of the items if a child did not have any needs in a specific category in the past 12 months. For example, if a child did not need to see a specialist in the last 12 months (answered “No” to CPR1), the child qualified as a legitimate skip for the question CPR2 that asked if there were any problems seeing a specialist.

### Statistical analysis

We randomly split the data into two halves. The first half (*training sample*, *n* = 8611) was used to identify promising items for comparisons among all possible short lists, and the second half (*validation sample*, *n* = 8779) was used to calculate all statistical measures reported in this paper. Ideally, the reported statistical measures should be based on data external to the development of the marker. In practice, split-sample development and validation sets are frequently used to develop instruments for screening diseases when a true external validation set from a unique sample is not available [21, 22], a limitation we have in this study.

For each half, we conducted a classification analysis using two-by-two contingency tables between children’s MH status and an item status, a domain status, or a marker status. Classification tests are a standard technique in clinical studies, where the two binary conditions consist of the true condition (e.g. sick/healthy) and a clinic diagnosis (e.g., at risk/no risk). Since all status variables are dichotomous, the classification test statistics is suitable in our study, where we treated the overall MH status as the true condition, and we used an item status, a domain status, or a marker status as a diagnosis.

The statistical measures in two-way clinic classifications include sensitivity, specificity, positive predictive value (PPV), negative predictive value (NPV), efficiency, and Cohen’s Kappa [23, 24]. The true positive (TP) cases were those children who were positive on the diagnosis status (e.g., an item status) and had a MH. The false positive (FP) cases included those children who also scored positively on an item or domain but did not have a MH. Among those children who had a negative item or domain status, the true negative (TN) cases and the false negative (FN) cases were defined with the former indicating children who had a negative item or domain status and did not have a MH and the latter indicating children who had a negative item or domain status but had a MH. Using these notations, we defined:

- PPV = TP/(TP + FP),
- NPV = TN/(TN + FN),
- Sensitivity = TP/(TP + FN),
- Specificity = TN/(TN + FP), and.
- Efficiency = (TP + TN)/N,
- where N = TP + TN + FP + FN is the total sample size.

We defined the positive rate of an item, domain, or marker as the proportion of children who had a positive status, i.e., (TP + FP)/N. Specificity, sensitivity, PPV, and NPV are conditional probabilities. Efficiency is the probability that a diagnosis agrees with the true condition, which can be less informative when the prevalence rate of the disease is too high or too low. To assess the probabilities, we adopted the general rule of thumb: a probability above 0.9 is considered as high, between 0.8 and 0.9 as moderately high, and moderate between 0.7 and 0.8.

We also calculated Cohen’s Kappa, which adjusted for the probability of agreement between two measures by chance. Kappa is usually between 0 and 1 with a Kappa above 0.6 considered as substantial agreement, and a Kappa value around 0.5 indicating a good level of agreement [25].

### Brief marker

We developed several brief markers using subsets of items that are strongly associated with the overall MH status. Hypothetically, a brief marker can reliably predict the MH status, and can be easier to implement in a survey than the full list of the items currently used in the literature. We considered two types of markers. The first type dropped the concept of domains and used between two and four items from Table 1. The marker score was defined as the average of all items in the marker. The overall MH status was positive if the marker score was greater than 75 points. The second type maintained the four domains—within each domain, one or two items mostly associated with the domain status were selected. For the second type, we first calculated each individual domain score. A domain status was considered positive if the average score of selected items within the domain was greater than 75 points. The overall MH status was positive if all four domains were positive. We enumerated all possible combinations among the 22 MEPS items in Table 1 for each type of markers. To select the best markers, we required specificity, sensitivity, NPV, PPV, and efficiency all greater than 0.7, and Kappa greater than 0.5.

### Disparities analyses

After identifying the items and domains with strong association with MH status for all children, we conducted stratified analyses by status of special health care needs and by racial/ethnic group. We identified children with special health care needs by adopting the method by prior research that used the MEPS data to provide detailed information about this group of children [26]. We included three racial/ethnic groups, Latinos, non-Latino Whites, non-Latino Blacks while other groups, such as non-Latino Asians, were not included due to small sample size.

## Results

### MH status

Among the 8779 children in the validation sample, 4279 (48.7%) had a MH.

### Statistical measures of the associations between individual items/domains and MH status

Due to the large sample size, all statistical measures had very small standard errors, and the 95% confidence intervals for all measures had a half-length no greater than 0.01. Thus, we omitted 95% confidence intervals in all tables to save space. Statistical measures between item status and MH status are reported in Table 2. The rate of positive status on each individual item was reported under the “positive rate” column. The positive rate was above 85% for most items, except for ACC1 (“Does the provider have office hours at night or on the weekend?”) and ACC2 (“Any difficulty accessing care after hours?”). The positive rate for ACC1 and ACC2 was 51 and 70%, respectively, indicating that only 51% of children’s providers had office hours at night or on the weekends, and 70% of children did not have difficulty accessing care after regular hours.

**Table 2:** Reliability of single survey item in predicting MH status

Domain and item	Specificity	Sensitivity	PPV	NPV	Efficiency	Kappa	Positive rate ^**a**^
**Accessibility**							
ACC1	0.74	0.77	0.74	0.78	0.76	0.51	0.51
ACC2	0.53	0.94	0.66	0.90	0.73	0.47	0.70
ACC3	0.10	0.99	0.51	0.87	0.53	0.08	0.94
ACC4	0.25	0.98	0.55	0.93	0.61	0.23	0.86
**Family centered**							
FAM1	0.10	1.00	0.51	0.95	0.53	0.09	0.95
FAM2	0.14	0.98	0.52	0.87	0.55	0.12	0.92
FAM3	0.24	0.88	0.52	0.67	0.55	0.12	0.82
FAM4	0.17	0.97	0.52	0.84	0.56	0.13	0.90
FAM5	0.25	0.93	0.54	0.79	0.58	0.17	0.84
FAM6	0.09	0.99	0.51	0.87	0.53	0.07	0.95
FAM7	0.09	1.00	0.51	0.96	0.53	0.09	0.95
**Comprehensive** ^**b**^							
CPR2	0.07	0.98	0.50	0.81	0.51	0.05	0.96
CPR4	0.03	1.00	0.50	0.95	0.50	0.03	0.98
CPR6	0.13	0.99	0.52	0.96	0.55	0.12	0.93
CPR8	0.07	0.99	0.50	0.92	0.52	0.07	0.96
**Compassionate**							
CPA1	0.09	1.00	0.51	1.00	0.53	0.09	0.95

^a^ This is the proportion of patients having positive status for an item

^b^ A legitimately skipped item was treated as having positive item status

Table 2 also showed that sensitivity and NPV were generally high for all the items. Nevertheless, except for ACC1 and ACC2, all items had very poor specificity (below 0.25) and PPV (below 0.55). This combination of extreme values in statistical measures (high sensitivity and NPV, and low specificity and PPV) reflected the fact that most children endorsed almost all the survey items except for ACC1 and ACC2. Further, PPV for all the items (except for ACC1 and ACC2) was close to the rate of having a MH among the study children (48.7%), suggesting that responding affirmatively to these items (except for ACC1 and ACC2) was only slightly better than a random guess of the overall MH status. In comparison, both ACC1 and ACC2 have relatively high PPV (0.74 and 0.66 respectively).

Overall, ACC1 and ACC2 had moderate to high values on all statistical measures. ACC1 had a sufficiently strong prediction power for the MH status (efficiency = 0.75, Kappa = 0.51), and ACC2 was only slightly weaker (efficiency = 0.73, Kappa = 0.47).

Table 3 reported the statistical measures between domain status and MH status. Sensitivity and NPV were all equal to 1 because the definition of MH requires a positive status in each of the domains. Due to the key role of two accessibility measures (i.e., ACC1 and ACC2) in predicting the MH status, the accessibility domain had the strongest association with the overall MH status (efficiency = 0.92, Kappa = 0.83). The family-centered domain had a weaker but notable association with the overall MH status (efficiency = 0.64, Kappa = 0.29), while the other two domains were not predictive with Kappa below 0.2.

**Table 3:** Reliability of domain status in predicting MH status

Domain	Specificity	Sensitivity	PPV	NPV	Efficiency	Kappa	Positive rate ^a^
Accessibility	0.84	1.00	0.85	1.00	0.92	0.83	0.57
Family centered	0.29	1.00	0.58	1.00	0.64	0.29	0.85
Comprehensive ^a^	0.17	1.00	0.53	1.00	0.57	0.17	0.91
Compassionate	0.09	1.00	0.51	1.00	0.53	0.09	0.95

^a^ This is the proportion of patients having positive status for a domain

A legitimately skipped item was treated as having positive item status

### Brief markers

Among those brief markers without domains that met the requirement in all statistical measures and had the highest Kappa, the selected 2-item marker included only ACC1 and ACC2. Adding one or two more items to it did not improve substantially the statistical measures, especially specificity. Of the markers with the four domains, either the 4- or the 7-item marker did not increase classification measures, compared with the above selected 2-item marker (Table 5 in Appendix).

### Disparities in the selected items and domains

Table 4 summarized disparities in the selected items and domain that had the strongest associations with MH status in Tables 2 and 3. For ACC1, while there were small differences between children with special health care needs and children without special health care needs, the differences were substantial across racial/ethnic groups. For example, the sensitivity of ACC1 in predicting MH status was 0.80 for Latino children, higher than that for either non-Latino Whites (0.71) or non-Latino Black (0.65). The positive rate of ACC1 was 0.42 for Latino children, markedly lower than that for non-Latino Whites (0.56) or non-Latino Black (0.59).

**Table 4:** Reliability of the selected items and domain in predicting MH status by special health care needs status and race/ethnicity

Selected Item/Domain	Specificity	Sensitivity	PPV	NPV	Efficiency	Kappa	Positive rate ^**a**^
**Item— ACC1**							
Special health care needs Status							
Yes	0.70	0.79	0.71	0.78	0.74	0.49	0.54
No	0.75	0.78	0.76	0.78	0.77	0.53	0.51
Race/Ethnicity							
Non-Latino white	0.71	0.80	0.76	0.75	0.76	0.51	0.56
Non-Latino Black	0.65	0.83	0.71	0.78	0.74	0.48	0.59
Latino	0.80	0.76	0.71	0.84	0.78	0.55	0.42
**Item— ACC2**							
Special health care needs status							
Yes	0.52	0.94	0.65	0.90	0.72	0.45	0.70
No	0.53	0.94	0.67	0.90	0.74	0.47	0.70
Race/Ethnicity							
Non-Latino white	0.46	0.95	0.67	0.88	0.72	0.42	0.76
Non-Latino Black	0.46	0.91	0.64	0.84	0.69	0.37	0.73
Latino	0.59	0.92	0.59	0.92	0.72	0.46	0.61
**Domain—Accessibility**							
Special health care needs status							
Yes	0.80	1.00	0.81	1.00	0.89	0.79	0.57
No	0.85	1.00	0.87	1.00	0.92	0.85	0.57
Race/Ethnicity							
Non-Latino white	0.80	1.00	0.86	1.00	0.91	0.81	0.64
Non-Latino Black	0.84	1.00	0.86	1.00	0.92	0.84	0.58
Latino	0.88	1.00	0.85	1.00	0.93	0.86	0.48

^a^ This is the proportion of patients in the subgroup having positive status for an item or domain

For ACC2 or the Accessibility domain, a similar pattern appeared with (1) small differences between children with special health care needs and children without special health are needs, (2) considerable differences across racial/ethnic groups, and (3) lower positive rate for Latino children, compared with non-Latino Whites or non-Latino Blacks.

## Discussion

Despite the efforts to promote MH in recent years, we found that only around 50% children nationwide had a MH, a finding that is consistent with the results from previous studies [6, 7]. Similar to prior research [17], this study found the family-centered care domain to be more predictive of MH status than the comprehensiveness domain. However, it is the accessibility domain that has the strongest association with the MH status. Thus, accessibility measures may be the most significant marker for predicting the MH status among pediatric patients. The top two items with the strongest association with the MH status were regarding doctors’ office hours at night or on the weekend and children’s difficulty accessing care after hours, highlighting the importance of after-hours primary care access for a well-functioning health care system, which has been documented by both international studies and prior research in the US [27, 28].

We also found that, while the two items and the accessibility domain did not differ significantly by status of special health care needs, there were considerable disparities in the two items and the accessibility domain across racial/ethnic groups with Latino children lagging behind other children. Similar findings were reported by prior studies. For example, one study also used the MEPS data and found small differences between children with special health care needs and children without special health care needs in terms of either the overall MH status or specific MH components [29]. On the other hand, like our analysis, prior research indicated lower likelihood of Latino children having a MH [30, 31].

This study is subject to a number of limitations. First, the definition of MH based on MEPS data can only cover four domains besides having a qualified USC. This is a limitation of the MEPS data as recognized in the literature [7, 8, 16]. Studies using other survey data, for example, the National Survey of Children’s Health, also included only three or four domains [17, 32]. It will be interesting to examine how the results might change if a comprehensive patient experience survey can cover all seven domains of the AAP definition of MH. Second, a large proportion of children in MEPS did not have complete information across the 22 survey items to define their MH status. Our findings may be subject to selection bias due to potential non-ignorable missing data. However, the sheer amount of missing data also strongly suggests the necessity and utility of a brief marker for children’s MH status, which may lead to less missing data in future studies, allowing for greater accuracy in ascertaining MH status for children. Third, although we used the split samples to develop and examine reliability measures of the markers, the split samples were retrieved from the same data source, and hence subject to the same sampling bias [33]. A truly external validation sample, independent of instrument development, is still needed for validation [21, 34, 35].

Despite these limitations, our analysis showed that accessibility is the most significant marker of children’s MH status. The high positive rates of all other items and domains make them less informative in predicting the MH status although they are equally important in measuring different perspectives of a MH. On the other hand, the relatively low positive rate of accessibility measures led to high predictive power, but also revealed a significant practical issue for children’s health care nationwide. Specifically, our finding that only 51% of children’s providers had office hours at night or on the weekends indicates that accessibility of health care after regular hours appears to be a major hurdle for pediatric patients to have a MH. The finding suggested that improving access to after-hours primary care services should be a priority for promoting the MH model for children.

Another implication of this study is to encourage a reconsideration of the effects of MHs on outcomes such as utilization, expenditures, quality of health care, and well-being outcomes. Given our findings, it is reasonable to suspect that accessibility to health care, particularly after regular office hours, may play an important role in producing these reported effects of a MH. One study found that among seniors, items in the accessibility domains affected various expenditure outcomes: no difficulty contacting providers over the telephone during regular business hours was associated with lower total and inpatient expenditures, and having a provider with office hours at night or on weekends was associated with lower outpatient, emergency department, and other expenditures [28]. While the precise nature of the relationship between accessibility measures and health care outcomes would benefit from further examination, one potential explanation is that not all MH domains are equal in practice, and among the domains of a MH, accessibility is likely the most difficult for a practice to achieve while also lying largely within a practice’s control. In comparison, measures in the comprehensive domain, such as specialist referrals, depend largely on patient-level factors and health care providers outside a primary care physician’s practice, making it difficult for the practice to directly affect changes in that domain. A patient-centered practice that considers the needs of patients and their families and works providers’ schedules around those of working parents and families may be more committed to improving accessibility and more likely to exhibit other features of a MH. Therefore, accessibility may not be a fundamental cause for conferring the benefits of a MH but serve as a critical mediator between practice features and the benefits of a MH. Future studies are needed to disentangle and confirm the effect of accessibility measures and the overall impact of having a MH on health care outcomes in the general pediatric population.

Finally, our finding of racial/ethnic disparities in the accessibility domain and the two accessibility items suggested that the ongoing efforts to promote MHs among children should target Latino children by increasing their access to care in order to improve their MH status.

## Conclusions

Less than half of children in the U. S had a MH. Among the survey domains, the accessibility domain had the strongest association with children’s MH status. The top two items with the strongest associations with the status ask about doctors’ office hours at night or on the weekend and children’s difficulty accessing care after hours. The two items can be used as a brief marker of children’s health status while combining them with more items will not necessarily improve the marker’s statistical measures. The significant associations between the two items and children’s MH status and the racial/ethnic disparities in these two items suggest that the ongoing efforts to promote the MH model need to target improving accessibility of health care after regular hours for children overall and especially for Latino children.

## Data Availability

The datasets analyzed by the current study are publicly available at the AHRQ web site (https://meps.ahrq.gov/mepsweb/data_stats/download_data_files.jsp).

## Appendix

**Table 5:** Reliability of top Type 1 (without domains) and Type 2 (with domains) Marker in predicting MH status

Marker	Items in marker	Specificity	Sensitivity	PPV	NPV	Efficiency	Kappa	Positive rate ^a^
**Type 1 without domain**								
2-item	(ACC1, ACC2)	0.86	0.71	0.76	0.83	0.79	0.58	0.41
3-item	(ACC1, ACC2, ACC4)	0.87	0.73	0.77	0.85	0.80	0.61	0.43
4-item	(ACC1, ACC2, ACC3, ACC4) ^b^	0.84	1.00	0.85	1.00	0.92	0.83	0.57
**Type 2 with domain**								
4-item	(ACC1, FAM5, CPR6, CPA1)	0.89	0.72	0.86	0.77	0.80	0.61	0.41
7-item	(ACC2, ACC4, FAM1, FAM5, CPR6, CPR8, CPA1)	0.75	0.85	0.76	0.84	0.80	0.60	0.55

^a^ This is the proportion of patients having positive status for a marker

^b^ This 4-item marker includes exactly all the items in the accessibility domain

## Abbreviations

AAP: American Academy of Pediatrics
FN: False negative
FP: False positive
MEPS: Medical Expenditure Panel Survey
MH: Medical home
PPV: Positive predictive value
NPV: Negative predictive value
TN: True negative
TP: True positive
USC: Usual source of care
